# Corona discharge induced snow formation in a cloud chamber

**DOI:** 10.1038/s41598-017-12002-5

**Published:** 2017-09-18

**Authors:** Jingjing Ju, Tie-Jun Wang, Ruxin Li, Shengzhe Du, Haiyi Sun, Yonghong Liu, Ye Tian, Yafeng Bai, Yaoxiang Liu, Na Chen, Jingwei Wang, Cheng Wang, Jiansheng Liu, S. L. Chin, Zhizhan Xu

**Affiliations:** 1 State Key Laboratory of High Field Laser Physics, Shanghai Institute of Optics and fine Mechanics (SIOM), Chinese Academy of Sciences, No. 390, Qinghe Road, Jiading District, Shanghai, 201800 China; 2 MOE Key Laboratory of Advanced Micro-structured Material, Institute of Precision Optical Engineering, School of Physics Science and Engineering, Tongji University, Shanghai, 200092 China; 3 IFSA Collaborative Innovation Center, Shanghai Jiao Tong University, Shanghai, 200240 China; 4 Center for Optics, Photonics and Laser (COPL), Laval University, Quebec City, QC G1V 0A6 Canada

## Abstract

Artificial rainmaking is in strong demand especially in arid regions. Traditional methods of seeding various Cloud Condensation Nuclei (CCN) into the clouds are costly and not environment friendly. Possible solutions based on ionization were proposed more than 100 years ago but there is still a lack of convincing verification or evidence. In this report, we demonstrated for the first time the condensation and precipitation (or snowfall) induced by a corona discharge inside a cloud chamber. Ionic wind was found to have played a more significant role than ions as extra CCN. In comparison with another newly emerging femtosecond laser filamentation ionization method, the snow precipitation induced by the corona discharge has about 4 orders of magnitude higher wall-plug efficiency under similar conditions.

## Introduction

Drought and desertification become a threat to human survival and development. Artificial rainmaking could help by improving the utilization of water resources up in the sky and bring about impressive socioeconomic benefits to societies^1^. The ionization method was first proposed in 1890 by Nikola Tesla for weather modification. Since then, most researchers aimed at using high voltage induced ionization/discharge to create a lot of supplementary cloud condensation nuclei (CCN) to induce precipitation^2–4^. However, mostly condensation was observed but with no precipitation^5^.

In this work, we report the first experimental observation of discharge induced artificial snowmaking in a cloud chamber. A static corona discharge was turned on inside a diffusion cloud chamber for about 25 min. between a pointed positively charged high voltage electrode and a grounded cold plate. Precipitation occurred in relatively large quantity. We attribute this phenomenon to the precipitation induced by corona induced ionic wind rather than by the ions as extra CCN. The ionic wind is essentially due to the acceleration of positive charges along the electric field lines originating from the pointed electrode towards the surface of the grounded plate. Collisions of these ions with air molecules would result in flows of air packets/parcels along the directions of the field lines.

## Results

**Figure 1:**
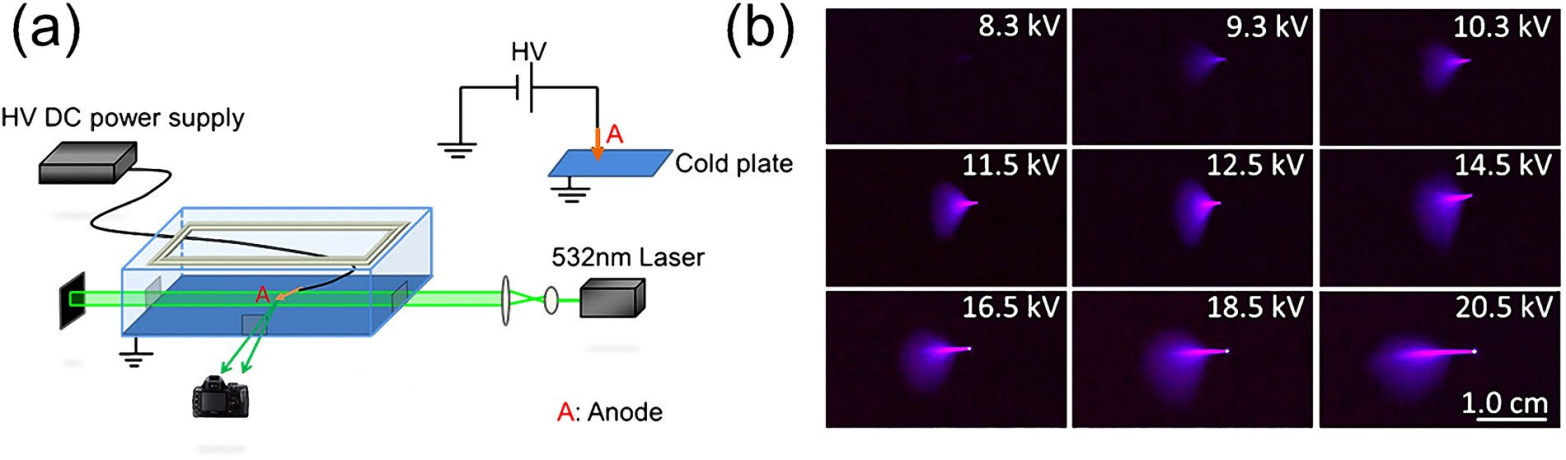
(**a**) A schematic diagram of the experimental setup. (**b**) Corona discharge under different high voltages. The electrode height was fixed at ~2.6 cm relative to the cold bottom plate.

**Figure 2:**
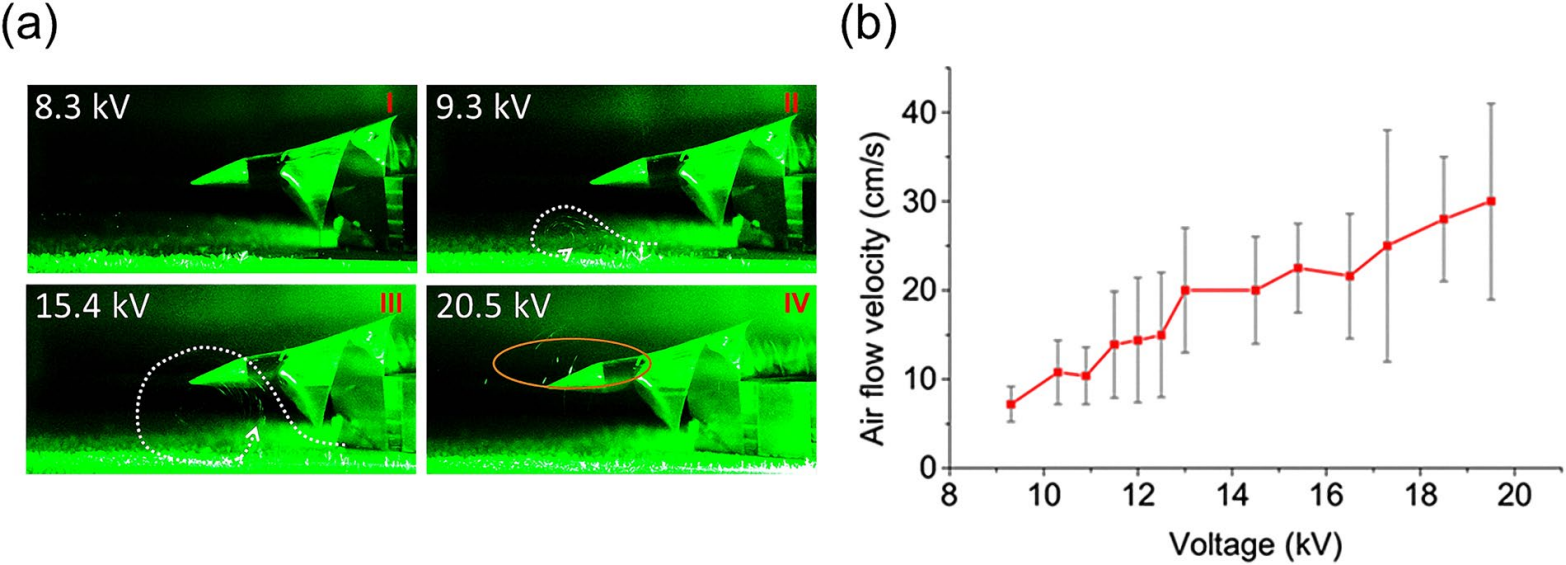
(**a**) Side images of corona discharge induced air flow at different high voltage on the electrode. The electrode was set at about 2.6 cm above the grounded cold bottom plate. White dotted lines indicated the moving directions of the air flow. (**b**) Airflow velocity vs. the voltage applied on the electrode.

The experiments were conducted inside a diffusion cloud chamber with the experimental setup schematically shown in Fig. 1a. As shown in the supplementary video, before the corona discharge was triggered, many background particles (ice and water droplets) floated around slowly with an average velocity ~3.0 cm/s and their maximum size was measured to be ~50 μm (Fig. 2a). Once the corona discharge was triggered, bluish-violet fluorescence emitted from the corona discharge was recorded (Fig. 1b, side view). The fluorescence was due to the ionization of nitrogen molecules and by collisional excitation^6,7^. Figure 1b shows that the fluorescence emission became stronger as the voltage was increased. This indicated that more plasma was generated around the tip at higher voltages.

An anti-clockwise vortex appeared within the slightly turbulent air below the electrode at ~2.6 cm from the grounded cold bottom plate when the voltage was increased to ~9.3 kV (Fig. 2a and supplementary video). The vortex was close to the cold plate with a diameter of ~1.5 cm. Packets of fog were formed and together with large size particles they followed the vortex motion below the electrode, with an average velocity of ~7.2 ± 2.2 cm/s. Generally, the particles moved away from the electrode and many of them ‘dropped’ quickly onto the grounded cold plate. This is an indication of the ionic wind following the electric field direction towards the ground. As the voltage increased, both the velocity and size of the vortex increased gradually (Fig. 2b and supplementary video). Packets of fog started to appear around the electrode at voltages higher than 14.5 kV. At 20.5 kV, the velocity of the particles reached up to 30 ± 13 cm/s (Fig. 2b). At the center of the vortex, the air flow ran much faster with a detectable particle velocity of ~75 cm/s (20.5 kV). Large size particles with diameters up to 200–300 µm were captured by the camera (as shown in the elliptical zone in Fig. 2A IV). Above 20.5 kV, mostly turbulent air was created. A maximum velocity of the large size particles dragged by the ionic wind reaching up to about ~1.0 m/s was estimated at 30 kV (with electrode height of 3.0 cm). Thermal effect of the plasma generated by the corona discharge would contribute to the vortex/turbulence motion through convection but not in a dominant way.

**Figure 3:**
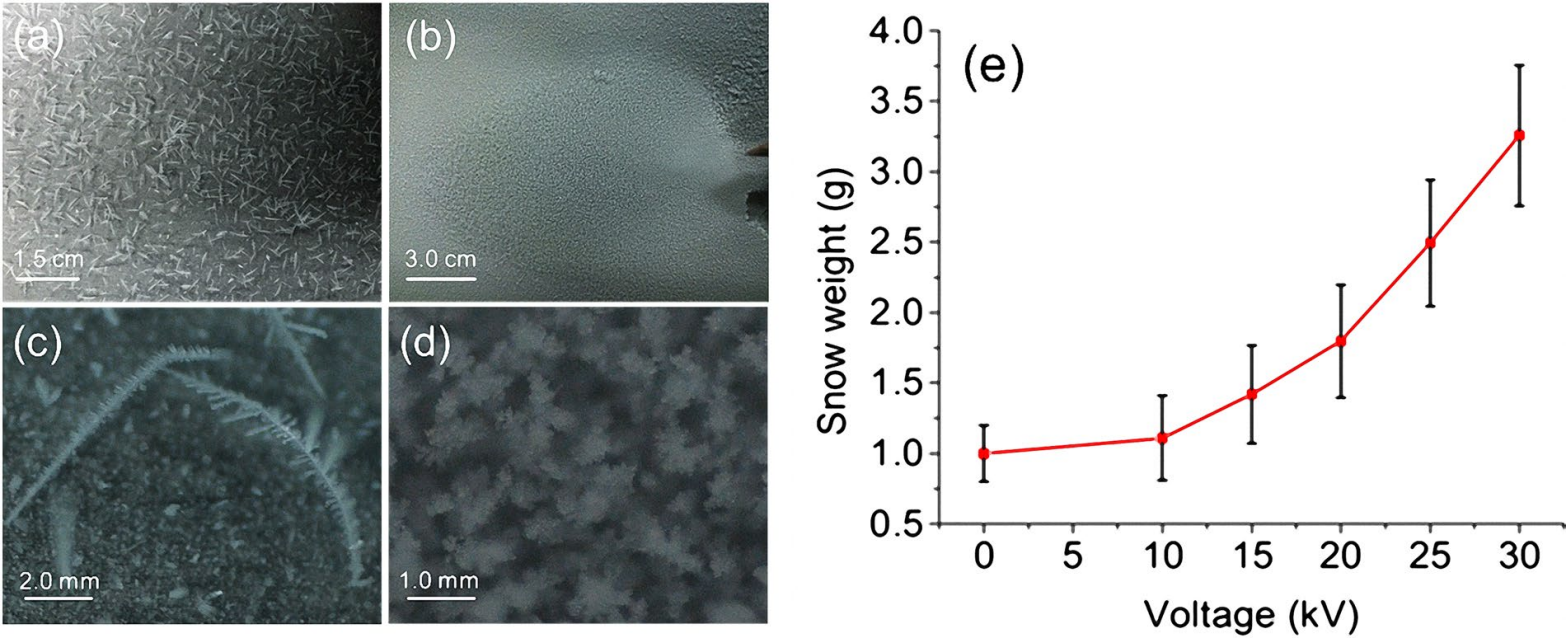
(**a**) Background snow formation on the cold bottom plate; (**b**) Snow formation on the cold plate when the corona discharge was turned on at 10 kV inside the chamber. (**c**,**d**) Close-up shots of typical snowflakes in background (**a**) and corona discharge-induced snow formation (**b**), respectively. (**e**) Weight of snow induced by corona discharge vs. the voltage applied on the electrode (with electrode height fixed of 3.0 cm).

The violent air flow motion around the electrode mixed the air up in the region between the electrode and the cold plate where there was a temperature gradient. At the end, instead of a uniform and loose distribution of background snowflakes on the cold plate (when no corona discharge was involved as shown in Fig. 3a), a thick, dense snow pile was observed below the electrode at 10 kV. It had a leaf-shape (Fig. 3b) very similar to the enlarged projection of the corona discharge onto the cold plate. It occupied principally an area of ~7.8 cm × 5.3 cm below the electrode. The snowflakes were more like ice particles and had an average size of ~0.9 ± 0.45 mm (Fig. 3d). This was much smaller than the average size of the background snowflakes (2.3mm ± 0.26 mm) (Fig. 3c). The size of the snow/ice particles close to the electrode was even smaller than that far away from the electrode.

The voltage on the electrode was varied from zero to 30 kV with the electrode rose a bit from the height of ~2.6 cm to 3.0 cm (in order to avoid breakdown at 30 kV). The total snow weight was measured by collecting all the snow covering the whole cold plate. As shown in Fig. 3e, the snow weight increased from 0.99 ± 0.45 g at zero volt (background) to 3.25 ± 0.5 g at 30 kV which was 3–4 times the background snow weight. Since both the snow weight (Fig. 3e) and the air flow velocity (Fig. 2b) increased with the applied voltage, one could conclude that the larger the air flow motion was, the more snow was formed.

**Figure 4:**
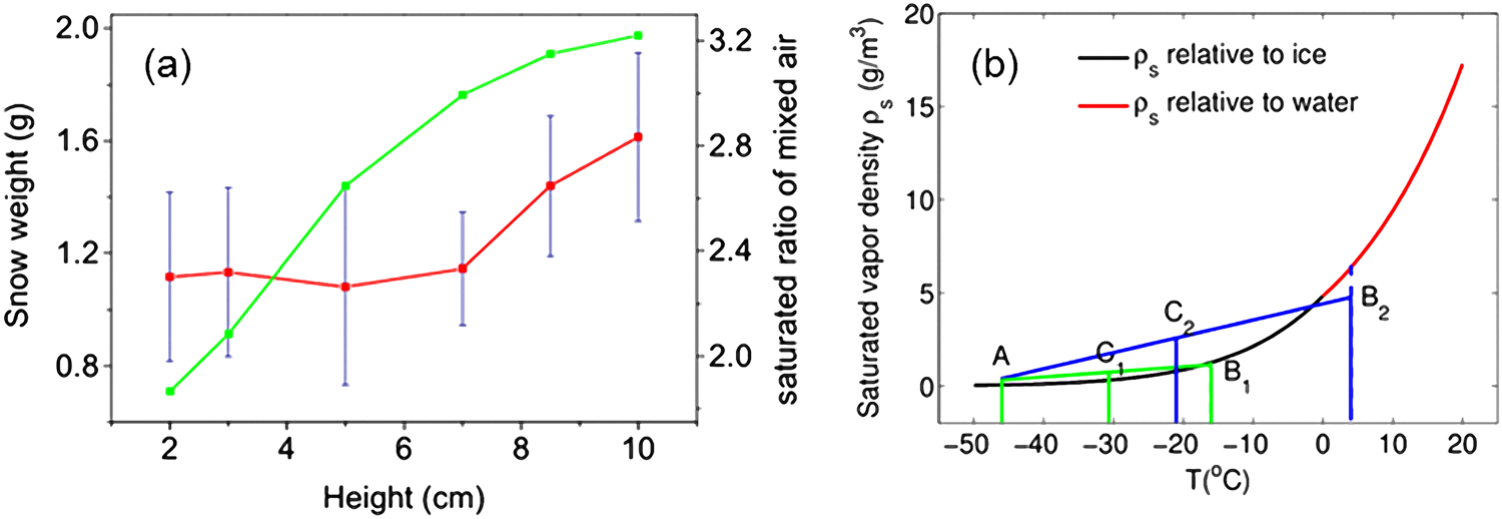
(**a**) Snow weight vs. different heights of electrode relative to the cold bottom plate (red curve) (the voltage applied was fixed at 10 kV) and the corresponding calculated saturation ratio of mixed air (green curve); (**b**) Saturated vapor density ρ_s_ of mixed air at representative heights of 2.0 cm (c_1_, green lines) and 10.0 cm (c_2_, blue lines).

The height of the electrode relative to the cold plate was also varied from 2.0 cm to 10.0 cm with the applied voltage fixed at 10.0 kV. The vertical temperature gradient from the electrode to the cold bottom became larger gradually. Vortex/turbulence always appeared between the electrode and the cold bottom plate. The velocity of the air flow dragged by the ionic wind was the same, due to the same static field intensity under fixed high voltage. When the electrode height was at 2.0 cm, the total snow weight was 1.12 ± 0.3 g (Fig. 4a ﻿the red curve). It increased gradually with the increase of the electrode height, even when the relative humidity around the electrode decreased. When the electrode was raised to a height of 10.0 cm, i. e. in the middle of the chamber, the snow weighed about 1.62 ± 0.35 g. The air with a temperature gradient between the electrode and the cold plate was mixed by the vortex/turbulence at different electrode heights. This would result in super-saturation (This is based upon an original idea of T. Leisner from the Karlsruhe Institute of Technology (Germany), in a private discussion with one of us (SLC) in 2013). The saturation ratio (which is defined as the real vapor density/saturated vapor density) was calculated using the same equation as given before^8^ (Fig. 4b). The saturation ratios for different electrode heights based on the saturation curve in Fig. 4b were presented as the green curve in Fig. 4a. It showed that the mixed air for different electrode heights was always super-saturated. The higher the electrode relative to the cold plate was, the higher was the saturation ratio, and the larger was the total snow weight.

## Discussions

The acidity of the snow induced by the corona discharge was measured roughly by pH test papers; it showed that the snow was almost neutral. To confirm this observation more precisely, the concentration of NO_3_^−^ in the snow was measured by an ion chromatograph (DionexICS-5000+); it was indeed very low, ~ 0.6 ± 0.05 ppm. This was compared to the NO_3_^−^ concentration ~0.2 ± 0.03 ppm in the background. One could conclude that little binary H_2_O-HNO_3_ was produced during the corona discharge. The CCN generated by the corona discharge would be mainly from the plasmas, which could charge the neutral molecules (such asO_2_^−^ etc.) and enhanced their ability of picking up water molecules around^9–11^. They grew up in size preferentially as a few large size big droplets and coagulated with other small droplets following the air flow motion stirred up mainly by ionic wind. However, there were already water/ice droplets and aerosols in the micrometer (μm) scale or larger (cloud) floating in the cloud chamber before the corona discharge was triggered. Such pre-existing droplets and aerosols would compete with the corona discharge generated ions, charged molecules such as O_2_^−^, and the little amount of binary chemicals such as HNO_3_ for the capture of water molecules and grow in size. Meanwhile, those pre-existing CCN and water/ice droplets would also collide with and adsorb the ions and chemicals rendering them even more active. We would thus expect that the pre-existing larger size CCN’s and water/ice droplets, including those activated by the corona induced ions and chemicals, would be the principal source for precipitation, i.e. the background particles were more important than the corona discharge induced charged molecules or chemicals^12^, especially the pre-exsting ice nuclei near the cold bottom plate. These background CCN and water/ice droplets would grow efficiently and precipitate in the sustained super-saturated environment which was achieved and maintained for a long period of time in the cloud chamber, through either coagulation or Wegener-Bergeron-Findeisen process.

From the experimental results and analyses presented above, we could conclude that to create precipitation in the present experiment, the following four conditions should be satisfied so as to create a sustained super-saturation environment: a high humidity environment, existing CCN, large temperature gradient and air mixing. These four conditions were also satisfied in our previous work by firing 1 kHz femtosecond laser pulses into the same cloud chamber^13–15^. Precipitation occurred in the filamentation case^13–15^. However, the weight of snow induced by filamentation was measured to be only about 13.0 mg under the highest available laser power (about 9 W) after 30 min. of irradiation^13^. This was more than 2 orders of magnitude less than the current case of about 2.25 g of net snow enhancement after applying 30 kV for 25 min. The electric power used in this corona discharge was very small, about 1–10 W in the present work (~0.27 mA with 20 kV applied). This power was similar to the optical power used in the femtosecond filamentation method. Moreover, by taking into account the wall-plug efficiency of femtosecond laser pulses which is usually less than 1% from electrical energy to optical energy, the overall wall-plug efficiency of corona discharge induced snow-making would be 4 orders of magnitude higher than that of the femtosecond laser filamentation method under very similar experimental conditions.

In summary, we demonstrated for the first time that a corona discharge could induce condensation and precipitation (snowfall). Precipitation occurred in relatively large quantity by turning on a static corona discharge inside a laboratory diffusion cloud chamber for 25 min. under a potential difference larger than 10 kV between a pointed electrode and the ground plate. We attribute this phenomenon to the precipitation in a sustained super-saturation environment aided mainly by the ionic wind from the corona discharge.

## Methods

### Experimental setup

The experiments were carried out in a laboratory diffusion cloud chamber of 0.5 × 0.5 × 0.2 m^3^ which was filled with ambient air. The cloud chamber had a bottom metal plate cooled down to ~−46 °C and a glass cover at the top which was kept at room temperature ~20 °C. Pure water was added into a rectangular water tank mounted about 17.5 cm above the cold plate inside the chamber. The chamber was sealed well and surrounded by thermal insulation foam to keep a stable temperature and humidity distribution inside. A copper cylindrical electrode (1.10 cm (bottom diameter) × 10 cm (length)) with a pointed end was fixed on a plastic base. The height of the electrode from the cold plate could be changed. The pointed end had a conic tip (1.1 cm (bottom diameter) ×1.6 cm (length)). The sharp tip had a diameter of ~0.5 mm. The electrode was connected to a high voltage DC power supply. The voltage applied on the electrode could be varied from 0 to 100 kV. The chamber was grounded safely by connecting the metal cold plate at the bottom to the ground directly. The electrode axis was parallel to the bottom base plate. A 2.0 W CW diode pumped semiconductor green laser was used as the probe beam propagating at the height of the electrode. In order to illuminate a large area, the probe beam was expanded in diameter and truncated by a 30 mm (height) × 5 mm (width) slit before passing into the chamber. The side Mie scattering was recorded by a digital single-lens reflex camera (Nikon D7000, 3264 × 4928 pixels) with a macro lens (AF 60 mm/2.8D). Inside the cloud chamber, the relative humidity and the typical vertical temperature distribution in the cloud chamber were measured with a ZDR-F20 Humidity Logger and a thermistor thermometer, respectively. From these measurements, the relative humidity and temperature at different heights relative to the cold plate which were used to plot Fig. 4 were obtained as given in Table 1.

**Table 1:** Relative humidity and temperature measured at different heights relative to the cold bottom plate inside the chamber.

Height	2.0 cm	3.0 cm	5.0 cm	7.0 cm	8.5 cm	10.0 cm
Relative humidity (%)	85.3	86.5	85.4	83.9	83.3	81.0
Temperature (°C)	−16.0	−13.0	−5.5	−1.0	1.0	3.5

In the present experiment, the total experimental duration was fixed at 50 min. During the first 25 min. the cloud chamber filled with ambient air was cooled down from room temperature to a stable state with a temperature gradient from −46 °C (bottom) to 20 °C (top). And in the last 25 min. corona discharge was triggered with the cooling on at the same time. At the end, the snow covering across the whole cold plate was collected into to a plastic tube, and weighed by an electric balance. Later the acidity of the collected snow was analyzed by pH test papers roughly, and also by a dedicated ion chromatograph (resolution 0.5 ppm) in the Shanghai Institute of Organic Chemistry (SIOC).

### Calculation of particle size and air flow velocity

To estimate the particle size, the effective size of each pixel of the CCD’s in the camera’s image plane was calibrated. At first, the scattering of the probe beam was recorded from the side view. Its real height was 30 mm as mentioned above. Secondly, we counted the number of pixels *n* that the image of the 30 mm high probe beam occupied. Considering that each pixel occupying a square area which means its height equals to the width, the effective size of each pixel was calibrated as 30/*n* × 30/*n* mm^2^. At the end, large size particles were chosen in the images taken from the side view. The number *m* of pixels occupied by a chosen particle was counted. Then the particle size was estimated as *m* × 30/*n* mm in diameter.

About the air current velocity, we disassembled the corresponding videos for side Mie scattering (25 fps) into individual frames. The distance L that the air current moved between two neighboring frames (with a time interval of *Δt* ~ 40 ms) was estimated and the air current velocity *v* was determined by *v* = *L*/*Δt*.

## Electronic supplementary material

Supplementary video

Supplementary information

## References

[1] Qiu J, Cressey D (2008). Taming the sky. Nature.

[2] Vonnegut B, Maynard K, Sykes WG, Moore CB (1961). Technique for introducing low-density space charge into atmosphere. J. Geophys. Res..

[3] Vonnegut B (1967). Technique for introduction into atmosphere of high concentrations of electrically charged aerosol particles. J. Atmos. Terr. Phys..

[4] Moore CB (1986). Abnormal polarity of thunderclouds grown from negatively charged air. Science.

[5] Reznikov M (2015). Electrically enhanced condensation I: effects of corona discharge. IEEE Trans. Industry Appl..

[6] Peek FW (1924). High-voltage phenomena. Jour. Franklin Inst..

[7] Wang T (2015). Direct observation of laser guided corona discharges. Sci. Rep..

[8] Ju J (2014). Laser-induced supersaturation and snow formation in a sub-saturated cloud chamber. Appl. Phys. B.

[9] Harrison RG (2000). Cloud formation and the possible significance of charge for atmospheric condensation and ice nuclei. Space Sci. Rev..

[10] Carslaw KS, Harrison RG, Kirkby J (2002). Cosmic Rays, Clouds, and Climate. Science.

[11] Kulmala M (2003). How particles nucleate and grow. Science.

[12] Dusek U (2006). Size matters more than chemistry for cloud-nucleating ability of aerosol particles. Science.

[13] Ju J (2012). Laser-filamentation-induced condensation and snow formation in a cloud chamber. Opt. Lett..

[14] Ju J (2013). Laser-filament-induced snow formation in a subsaturated zone in a cloud chamber: Experimental and theoretical study. Phys. Rev. E.

[15] Ju J (2016). Femtosecond laser filament induced condensation and precipitation in a cloud chamber. Sci. Rep..

