# Supplementary material for: Corona discharge induced snow formation in a cloud chamber
Source: Sci Rep. 2017 Sep 18;7:11749. doi: 10.1038/s41598-017-12002-5 (PMC5603531; doi:10.1038/s41598-017-12002-5)
Supplement: Supplementary file 2 — Supplementary information [file 41598_2017_12002_MOESM2_ESM.pdf]

Corona discharge induced snow formation in a cloud chamber

Jingjing Ju[1†], Tie-Jun Wang[1†], Ruxin Li[1*], Shengzhe Du[1], Haiyi Sun[1], Yonghong Liu[1,2], Ye Tian[1], Yafeng Bai[1], Yaoxiang Liu[1], Na Chen[1], Jingwei Wang[1], Cheng Wang[1], Jiansheng Liu[1,3], S. L. Chin[4], and Zhizhan Xu[1*]

[1]State Key Laboratory of High Field Laser Physics, Shanghai Institute of Optics and fine Mechanics (SIOM), Chinese Academy of Sciences, No. 390, Qinghe Road, Jiading District, Shanghai 201800, China.

[2]MOE Key Laboratory of Advanced Micro-structured Material, Institute of Precision Optical Engineering, School of Physics Science and Engineering, Tongji University, Shanghai 200092, China.

[3]IFSA Collaborative Innovation Center, Shanghai Jiao Tong University, Shanghai 200240, China.

[4]Center for Optics, Photonics and Laser (COPL), Laval University, Quebec City, QC G1V 0A6, Canada.

*Correspondence to: ruxinli@mail.shcnc.ac.cn, zzxu@mail.shcnc.ac.cn

†These authors contributed equally to this work.

Supplementary video information

Video 1: Air flow motion induced by corona discharge in a cloud chamber, with high voltage applied on the electrode being varied from 0 to 20.5 kV. The electrode was set horizontally at a height of ~2.6 cm relative to the cold plate.
